# Using evidence, expert opinion and epidemiological model to understand pathways to survival and mortality: The Pathways to Survival (PATHS) Tool

**DOI:** 10.7189/jogh.11.15001

**Published:** 2021-06-12

**Authors:** Igor Rudan, Evropi Theodoratou, Kit Yee Chan, Davies Adeloye, Ozren Polašek, Harry Campbell, Mickey Chopra

**Affiliations:** 1 Centre for Global Health, The Usher Institute, University of Edinburgh, Edinburgh, UK; 2 Croatian Centre for Global Health, University of Split School of Medicine, Split, Croatia; 3 Health Nutrition and Population, The World Bank, Washington, DC, USA

## Abstract

**Background:**

The reasons why episodes of illness can lead to fatal outcomes in affected persons in low resource settings are numerous and complex. A tool that allows policy makers to better understand those complexities could be useful to improve success of programmes that are implemented globally to reduce mortality.

**Methods:**

We developed a “Pathways to Survival” (PATHS) tool: an epidemiological model using decision trees, available evidence and expert opinion. PATHS visualises the “architecture” of mortality in the population by following the entire population cohort over a certain period of time. It explains how initially healthy persons progress through health systems to lethal outcomes at the end of the specified time period. We developed an illustrative example based on the 136 million newborns and an estimated 907 000 deaths from newborn sepsis in the year 2008. This allowed us to develop an epidemiological model that described pathways to deaths from neonatal sepsis globally in 2010.

**Results:**

The model described the “status quo’ situation in 2010 with 907 000 deaths to allow an assessment of the potential impact and feasibility of different interventions and programmes at various level of health systems in reducing this cause of mortality. A useful model should incorporate both a ‘horizontal’ and a ‘vertical’ component. The ‘horizontal’ would track the progress of all neonates globally through time, ie, their first 28 days of life, and separate them into different ‘pathways’ every time a change in their risk of dying from neonatal infection occurs because of their specific contextual circumstances. The ‘vertical’ would track their position within the health systems of their countries and separate them into different categories based on the ability of health system to intervene and reduce their risk of dying. Based on those requirements, PATHS tool was developed which is based on decision trees where different “branches” of the trees are associated with varying case-fatality rates.

**Conclusions:**

The application of the PATHS tool on the example of newborn sepsis revealed that novel diagnostic tests could save many lives, so we should continue to invest in them to improve their validity, deliverability and affordability. However, PATHS showed that investments in better diagnostics have limited impact unless they are coupled with improvements of the context. Programs for parental education improve compliance and care seeking. Promoting legislation change to empower community health workers (CHWs) to actively engage in prevention, diagnosis and care also makes a difference, as well as programs for training CHWs to use diagnostic tests and administer treatments correctly. Care-seeking behaviour can also be improved through programs of conditional cash transfers. Finally, PATHS demonstrated that improving access to primary and secondary health care for everyone is the most powerful contextual change.

The reasons why episodes of illness can lead to fatal outcomes in affected persons in low resource settings are numerous and complex. They often extend beyond the clinical course of disease: a so-called “social autopsy” may be just as important as the biological causes and effects of pathogens. The capacity of health systems to prevent, detect and treat illnesses is also a major component of the outcome. A tool that allows policy makers to better understand and map those complexities could be useful to ensure success of programmes that are implemented to reduce mortality in low-resource settings.

In this paper, we present a “Pathways to Survival” (PATHS) tool: an epidemiological model using decision trees, available evidence and expert opinion that can explain the “architecture” of mortality in the population. It achieves this through following the entire population cohort over a certain period of time and explains how initially healthy persons progress through health systems to lethal outcomes at the end of the specified time period.

We developed an illustrative example based on the 136 million newborns and an estimated 907 000 deaths from newborn sepsis in the year 2008 [1]. We gathered all evidence available in the year 2010, when this estimate was published, and we consulted the experts for further input [1]. This allowed us to develop an epidemiological model that described pathways to deaths from neonatal sepsis globally in 2010. At that point, there was little that could be done to prevent and treat neonatal infections in low-resource settings apart from early recognition of disease and rapid implementation of antibiotic treatment [2-4]. Another challenge was that in small neonates treatment usually needs to be parenteral, and the potential role of vaccination is limited by their undeveloped immune systems [2,3]. Clearly, the capacity of health system to establish early diagnosis, encourage care seeking behaviour among parents, and provide access to health care facilities where appropriate antibiotic treatment can be administered are the key requirements for a successful programme that aims to reduce mortality from neonatal infections. This is what made this example appealing, as it may have similarities to a number of other urgent conditions that require health systems' response in later life.

Approaches to reduction of mortality from neonatal infections globally in 2010 were limited by the very first step in this proposed process: a failure to diagnose them accurately in low-resource settings [5]. Most of the diagnostic tools were developed for the contexts of industrialized countries. Their implementation in low-resource settings was prevented by many factors. They included high cost of diagnostic tools, complexity and infrastructure requirements beyond capacity typically available in low resource settings, under-trained health care workers who administer the tests, low acceptability among parents, lack of quality control, and others [3,5]. Therefore, in low resource settings, the identification and management of newborn infections depended on clinical algorithms that incorporated signs and symptoms with modest sensitivity and specificity, which leads to both over- and under-treatment [3,5].

Point-of-care diagnostic tests were needed in low resource settings to diagnose neonatal infections, identify responsible pathogens, predict severe course of illness, choose appropriate treatment, monitor the effectiveness of antibiotics and document antibiotic resistance [6,7]. These tests, both the existing and emerging ones, could potentially be applied at different levels of health system, which mainly depends on their complexity and laboratory requirements. They could be “over-the-counter” tests which could be used by concerned parents, but also tests distributed to community health workers for population-based screening and early referral programmes, tests at the primary health care facility, or hospital-based tests [6,7].

We believe that one of the key obstacles to consideration of point-of-care diagnostic tests as potentially feasible approach among the key stakeholders in global health policy development is the absence of information about their potential effectiveness in mortality reduction. Indeed, unlike most other interventions that directly prevent or treat illnesses and reduce mortality, it is difficult to conceptualize the impact of point-of-care diagnostic tools on mortality reduction. Their role is indirect, but they can still help prevent deaths by changing the “status quo” situation. By providing timely information about the illness, which would not exist in their absence, point-of-care diagnostic tools can remove a neonate from a pathway that would lead to high risk of death and direct the child into an alternative pathway, where the risk of death would be much lower [8].

We realized that, if we could develop an appropriate epidemiological model – using the best available evidence and expert opinion – which would describe pathways to neonatal infections deaths under the current (“status quo”) situation, then it should be possible to quantify the reduction in the expected number of deaths that could be achieved through application of a point-of-care diagnostic tool at different levels of health system. In this way, both effectiveness and cost could be assigned to point-of-care diagnostics, and this would make them comparable with other interventions with known cost-effectiveness. Therefore, the main conceptual aim of the proposed PATHS tool is to allow an assessment of cost-effectiveness in relation to mortality reduction of an intervention such as point-of-care diagnostic test, which essentially provides information, rather than treatment, but the correct information can still save lives. The PATHS tool allows comparisons between all types of interventions in health care planning, including the implementation of diagnostic tests.

## METHODS

### Basic structure of the epidemiological model – a “horizontal” component

The experts who developed this model agreed that, in order to understand the potential effectiveness of the new diagnostic tests on neonatal sepsis mortality, we need to follow all neonates born within one year in the world “horizontally”, as they progress through the first 28 days of life. We also need to “branch” this progress and separate them into different ‘pathways’ every time a change in their risk of dying from neonatal infection occurs because of their personal circumstances. This would result in a horizontally laid “decision tree” in which different neonates follow different “branches” (pathways), each one of them carrying different risk of mortality from neonatal infection. However, the key challenge would be to understand how many neonates pass through each those branches each year globally, and what could be done at different levels of this “decision tree” to divert them from one pathway to another using information provided by a point-of-care diagnostic test. Application of the test should ensure that more neonates are diverted into branches with lower risk of dying, and less of them are remaining on pathways to higher mortality risk.

However, it has been noted that we also need to know the distribution of neonatal sepsis deaths by place in health system where they occur: ie, how many deaths are untreated and occur at home because neonates either do not seek care, do not have access to care, or their parents do not accept care; then, how many deaths occur after treatment is provided by a community health worker (CHW), at the level of primary health care facility (PHC), or within secondary health care facility (SHC). Once an infection occurs, the position of neonates within health systems of their countries will significantly affect their risk of dying, based on the ability of health system to reach them and provide effective treatment.

All the experts eventually agreed that a useful model should incorporate both a “horizontal” and a “vertical” component. The ‘horizontal’ one would track the progress of all neonates globally through time, ie, their first 28 days of life, and separate them into different “pathways” every time a change in their risk of dying from neonatal infection occurs because of their personal circumstances. The ‘vertical’ one would track their spatial circumstances, ie, their position within the health systems of their countries, and separate them into different categories based on the ability of health system to intervene and help them.

**Figure 1:**
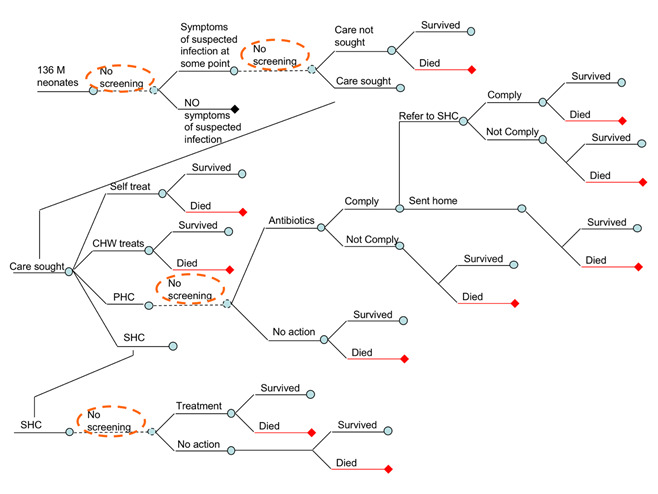
Basic epidemiological model showing possible pathways to deaths from neonatal infections globally and four points of possible interventions with diagnostic tests. CHW – community health worker; PHC – primary health care physician; SHC – secondary health care.

**Figure 1** shows the expert’s consensus on the basic structure of the epidemiological model. The logic of horizontal branching is reflecting any change in mortality risk of the neonates. The ‘tree’ starts, on the top of the left hand side, with 136 million neonates who were born globally in 2010. The first branching occurs when there is a sign or a symptom of suspected infection. Clearly, neonates with no signs or symptoms (the bottom branch following the second node) will not be under risk of dying from infection, because we assumed that each death would always be preceded with at least some sign or symptom of suspected infection. Those who develop signs/symptoms of suspected infection during neonatal period should be separated in the upper branch of the tree, following the second node. This pathway then branches further, depending whether the parents choose to seek care or not. This is because, all other things being equal, those who seek care are expected to reduce the risk of dying of their neonate in comparison to those who do not. Having agreed on this, we also recognized that the assumption of all other things being equal between non-care seekers and care seekers is a major assumption. Those with more severe presentation, and those with more highly educated parents, wealthier parents, and who live closer to health care facility will be more likely to seek care. This is further discussed in assumptions of the model (see next subheading).

**Figure 1** further shows that those who do not seek care may survive or die, while those who seek care may eventually reach different level of health system, depending on their access to care, the advice given by CHWs and primary care physicians, their compliance with this advice, their resources to pay for travel to higher-level facility, and the severity of their child’s illness. Eventually, care-seekers may end up self-treating the child, receiving care from CHWs, PHC physicians, or reach the hospital (**Figure 1**). The model is only interested in the highest level of care obtained. Self-treatment or treatment provided by CHWs have no further branching – the outcome is either survival or death from infection.

In an attempt to simplify the model, some experts questioned the reasons for splitting the pathway of those who self-treat, or receive treatment from CHWs, from those who do not seek any care. We have two justifications: first, we may assume some limited effectiveness of treatment attempts by non-health professionals, which reduces the risk slightly in comparison to those neonates to whom no care was offered; second, if point-of-care diagnostic tests became available, the parents who seek care either from CHWs or through self-treatment would be considerably more likely to accept testing and comply with its results, in comparison to those parents who do not seek care at all. The lack of care seeking can be caused by a number of reasons, including (but not limited to) lack of access, failure to recognize symptoms, insufficient funding and other context-specific and cultural reasons.

The next branch in **Figure 1** shows parents who reach primary health care facility, where we assumed that at least some antibiotic treatment would be available. However, the attending physician may decide that clinical algorithm for the diagnosis of neonatal infection does not provide sufficient indication for antibiotic treatment, thus sending the parents and the child back to their home (**Figure 1**, lower branch). There, the child may get well and survive, or they may die if physicians made an incorrect diagnosis. In cases where physicians provide antibiotic treatment (**Figure 1**, upper branch), the parents may either comply or not comply. Although the lower branch (‘non-compliance’) is unlikely among parents who initially sought care, we decided to leave it in the model to account for this theoretical possibility in cases where PHC physicians or nurses provide active community-based surveillance for neonatal infections, and where non-compliance may become an issue. The final branch at the primary health care level follows the fate of those neonates who received antibiotic treatment. They may be released home following the treatment, or be told by a concerned physician to proceed and seek care at the nearest hospital. Those who comply with this advice and can afford the trip will join other parents who went to hospital directly (**Figure 1**).

This creates a mixture of as many as four different groups of neonates seen at the hospital level (**Figure 1**): (i) some will present there regardless of the severity of their symptoms, simply because the access to hospital care is easy for their parents; (ii) others will be brought directly to hospital by those parents who were the most concerned, usually because of very severe symptoms, and these children bypassed all other levels of care; (iii) the third group will consist of children who already received antibiotic treatment at the PHC level, but the physicians were sufficiently concerned to refer them to SHC facility, and the parents complied with the suggestion; (iv) finally, the fourth group is also defined as the neonates born in hospital who were never released home after birth because of a suspected infection (see rationale for this later). The first group will have the lowest risk of dying, the second group the highest, the risk in the third group will probably somewhat lower than in the second group (because treatment has been initiated early), and the fourth group will have intermediate risk (early initiated treatment will reduce the risk, but the etiological spectrum of sepsis in the first 3-5 days of life is more life-threatening). The diagnostic procedures at the hospital will lead to the decision to either treat or release without treatment, and in both branches there will be neonates who will either survive, or eventually die from neonatal infection (**Figure 1**).

### Points for intervention using point-of-care diagnostic tools – a “vertical” component

The previous sub-heading explained the basic structure of the epidemiological model in terms of its ‘horizontal’ component. However, in order to understand the possible points of intervention using point-of-care diagnostic tool we also need to consider a ‘vertical’ component – ie, where do infected neonates find themselves within the health system once they develop symptoms of infection, and what is the highest level of care accessible to them. **Figure 1** shows the four main points of possible intervention from health systems’ perspective. At those four points, designated as ‘no screening’ in **Figure 1** (implying that under the current ‘status quo’ situation no point-of-care diagnostics are routinely available), information obtained from a highly sensitive and specific diagnostic tool could change the fate of the neonates who present themselves to that particular point of care. Based on the information obtained through the use of diagnostic tool those neonates could be ‘re-directed’ into another pathway, in which their risk of mortality would be lower. In this way, the point-of-care diagnostic tool would reduce mortality from neonatal infections, albeit indirectly, and this reduction could be quantified.

**Figure 1** shows that, conceptually, the four key points where diagnostics could be introduced are: (i) diagnostic testing (‘screening’) of seemingly healthy neonates for signs of invasive bacterial infection immediately after birth, and then subsequently throughout the neonatal period; (ii) diagnostic testing of those neonates who developed symptoms or signs of neonatal infection at the community level, either by parents or community health workers; (iii) diagnostic testing of neonates with suspected infection who sought care at the level of primary health care; and (iv) diagnostic testing of neonates with suspected infection who sought care at the level of secondary health care. Clearly, each of these four points for potential diagnostic testing would have its own characteristics in terms of skill and capacity of implementers of the test, feasible complexity and affordable cost of the test, and supporting laboratory requirements. The four points present a spectrum, in which community-based diagnostic screening would require very low cost and complexity of the test, very low level of skill of the implementers, and no laboratory requirements. At the other end of the spectrum, hospital-based tests could potentially have somewhat higher cost, greater complexity, and allow for greater skill required from the implementers and some laboratory requirements.

Finally, once that ‘horizontal’ and ‘vertical’ component of the model has been defined, we still needed to identify the potentially useful characteristics of a diagnostic test itself. The key question posed to the experts was: what information should a diagnostic test ideally provide at each of the four different points of care defined above to reduce the risk of mortality among the tested neonates? The experts agreed that, conceptually, there would be three general tiers of diagnostic tests of potential interest, and only some would be useful at each of the four points of care.

The **first type** of point-of-care diagnostic test would need to be able to identify the presence of invasive bacterial infection in a neonate with suspected infection. This test would require high degree of sensitivity and specificity, and its usefulness would be in establishing an indication for immediate antibiotic treatment. This test would be useful at all four levels of health systems – community level (as a screening among healthy neonates or intervention among possibly infected neonates), primary health care level and hospital care. This test would also empower community health workers to apply antibiotic treatment, which could dramatically expand coverage of community case management with oral antibiotics – proven to be effective even when implemented by CHWs [9,10].

The **second type** of diagnostic test should be able to identify the exact bacterial (or viral) pathogen that caused the infection. This information would prevent treatment with antibiotic which would not be effective against that specific pathogen, or which may fail to cure an infected neonate due to antibiotic resistance. Clearly, this type of test would not be particularly useful for screening of healthy neonates, or even for testing the neonates with suspected infections at the community level. This is because it is unlikely that CHWs would have the spectrum of low-cost and higher-cost antibiotics available to them in the present global context, and also necessary skill to apply them properly based on test results. However, this test may be very useful to a PHC-level physician, who may have a wider choice of antibiotics, and whose rapid and effective response to neonatal infection with appropriate and effective antibiotic may indeed save lives. Similarly, this test could also be useful at the hospital level, especially among severe cases who require intensive care and where a rapid and effective treatment is critical.

The **third type** of diagnostic test should imply that the episode is likely to become severe and require hospital care. This information would be expected to not only indicate immediate antibiotic treatment, but also prompt care seeking directly at the level of hospital care. The experts debated whether this test would really be useful, or whether any invasive bacterial infection among neonates should be considered life threatening and require hospital referral. Eventually, they agreed that it would be useful to have a test that implies development of a severe or very severe episode, because those children are most likely to die and rapid transfer to hospital-level intensive care unit could perhaps avert many of those deaths. With respect to the level of health system at which this test could be useful, the experts agreed that this test would not be feasible for screening of healthy neonates because they would be unlikely to appear healthy if they had biochemically detectable signs of severe or very severe infection. The test could be useful for those with suspected infection at the community level, because it could make a difference whether a CHW would suggest referral to PHC or SHC. It would also be useful to PHC physicians, to help them decide whether to suggest further hospital referral. It could be used in the hospitals to assist triage procedures when assigning sick neonates to intensive care units.

### Definitions and assumptions underlying the proposed epidemiological model

The PATHS tool is an epidemiological model based on decision trees that describes pathways to deaths in a defined population. In this particular example, the population are world's neonates in one year and the deaths are caused by neonatal infection. Therefore, we first need to define what do we mean by ‘neonatal infection’. The experts agreed that these should actually be termed more precisely – as “suspected potentially life threatening invasive bacterial infections” (SPLTIBI). This definition obviously excludes all skin infections, tetanus, diarrhoea, malaria, HIV/AIDS and other non-invasive or non-life threatening infections in neonates. For the ease of presentation, however, we will simply continue to refer to SPLTIBI as ‘neonatal infections’ in further text.

An important suggestion from the experts was that, given that the etiological spectrum and severity of neonatal infections differ considerably between the first 3 days of life and the period between 4-28 days of life, and that the number of neonatal deaths globally are split almost equally between those two categories, perhaps we need two separate epidemiological models – for “early” vs “late” neonatal infections? After discussing this issue at some length, the experts agreed that fundamentally the severity of infection and reaction of the parents/carers will have similar consequences in both time periods, and that the pathogens that can be responsible for infection are not exclusively found in only one period – they are just more likely. Therefore, we agreed that early and late infections are not clearly separate entities from the etiological perspective and we agreed not to develop two separate models. Finally, as it will become apparent in later text, the information available for different model parameters was very sparse. Therefore, introducing an additional separation in the model would produce less reliable estimates. For similar reasons (lack of specific information), we chose not to separate neonates according to their place of birth, birth weight and other risk factors that might affect the development, progression and outcome of neonatal infections.

For similar reasons, we made no distinction between “early vs. late” sepsis, “advanced vs. non-advanced” sepsis, or “sepsis vs. severe sepsis”. In the early versions of the model we had two defined steps in the sepsis progression: sepsis and severe sepsis. We assumed that all sepsis deaths will derive from the severe sepsis phase. However, the experts at the third TAG meeting advised that the severe sepsis step is not necessary because sepsis is a very severe disease and that a high proportion of sepsis cases will lead to death if left untreated. This is why we considered all cases of neonatal sepsis as a dangerous and life-threatening condition which requires treatment and hospital care. Also, we used ‘no action’ abundantly in the model, although this term may not be strictly correct, because there’s usually always some action at some stage, although it may be too late to change the course of the disease.

All hospital births after which sepsis was suspected at hospital and that were not released home for this reason were presented as “seeking care at the SHC level”, because this was much simpler to present and easier to understand. This approach essentially defines parameters 6-9 in **Table 1** as ‘the highest level of received care’. Also, the experts agreed that parameters 14-21 in **Table 1** are all needed, because diagnosis at home by parents, by CHW, or by doctors at the PHC or SHC level all have their different sensitivity and specificity. Similarly, the treatments that they provide will have different effectiveness, as captured by parameters 10-13 in **Table 1**. The experts argued whether sensitivity at the PHC level may increase due to multiple visits, but this showed not to be of sufficient importance to the model's output to justify a special consideration.

**Table 1:** List of parameters that were used in our proposed epidemiological model of pathways to deaths from neonatal infections globally

Parameter number	Definition	Value	Source
1	Number of neonates	136 241 000	World Health Organization (www.who.int)
2	Proportion of neonates with symptoms of suspected infection	0.12	Delphi exercise among technical experts at the meeting in September 2011
3a	Proportion of neonates with symptoms of suspected infection that sought care	0.69	Chandran et al., Herbert et al. systematic reviews [11,12]
3b	Proportion of neonates with symptoms of suspected infection that didn't seek care	1 – 0.69 = 0.31	1 – proportion of neonates that sought care
4	Proportion of neonates with symptoms of suspected infection that didn't seek care and developed sepsis	0.087	Based on the analogy with childhood pneumonia [13,14]
5	CFR of untreated severe sepsis	0.80	Delphi exercise among technical experts at the meeting in September 2011
6	Proportion of neonates with symptoms of suspected infection that sought care: Self treatment	0.16	Chandran et al., Herbert et al. systematic reviews [11,12]
7	Proportion of neonates with symptoms of suspected infection that sought care: CHW treatment	0.15	1-proportion of neonates that sought care at home, in PHC or in SHC
8	Proportion of neonates with symptoms of suspected infection that sought care: PHC or medically trained provider	0.37	Chandran et al., Herbert et al. systematic reviews [11,12]
9	Proportion of neonates with symptoms of suspected infection that sought care: SHC	0.32	Chandran et al., Herbert et al. systematic reviews [11,12]
10	Effectiveness of home care interventions	0.10	Delphi exercise among technical experts at the meeting in September 2011
11	Effectiveness of CHW interventions	0.15	Delphi exercise among technical experts at the meeting in September 2011
12	Effectiveness of PHC interventions	0.35	Delphi exercise among technical experts at the meeting in September 2011
13	Effectiveness of SHC interventions	0.58	Delphi exercise among technical experts at the meeting in September 2011
14,15	Sensitivity and specificity of home care treatment	0.50, 0.50	Delphi exercise among technical experts at the meeting in September 2011
16,17	Sensitivity and specificity of CHW treatment	0.60, 0.60	Delphi exercise among technical experts at the meeting in September 2011
18,19	Sensitivity and specificity of clinical algorithm at PHS to detect sepsis	0.70, 0.70	Delphi exercise among technical experts at the meeting in September 2011
20,21	Sensitivity and specificity of clinical algorithm at SHC to detect sepsis	0.90, 0.70	Delphi exercise among technical experts at the meeting in September 2011
22	Proportion of parents that comply with antibiotics prescription from PHC doctor	0.90	Delphi exercise among technical experts at the meeting in September 2011
23	Proportion of parents that comply with referral to SHC from PHC doctor	0.60	Delphi exercise among technical experts at the meeting in September 2011

CHW – community health worker; PHC – primary health care physician; SHC – secondary health care; CFR – case-fatality rate

Also, we treated each episode of suspected sepsis as an individual one, with no “backward loops” of multiple episodes per neonate. This is because a possible increased incidence risk and/or case fatality ratio (CFR) of neonates with multiple sepsis episodes is reflected in the overall CFRs. Essentially, multiple episodes don’t matter because the affected neonates must survive all previous episodes to experience the last one that occurred within the first 28 days of their life. We also assumed that there would be no increase in the effectiveness of interventions for neonates that visited more than one type of point of care. Therefore, the effectiveness of intervention for each neonate was the one provided at the highest point of care. This is particularly relevant to neonates who received antibiotic treatment at the PHC level but still proceeded to seek care at the CHC level. We assumed that PHC doctors referred all the cases of sepsis that they recognised based on their clinical algorithm to a SHC facility after one course of treatment.

Experts suggested that one arm should perhaps follow the neonates treated with oral, and another one with parenteral antibiotics. However, others stated that trials show very high effectiveness with either one of those two treatment types, albeit this is actually a comparison between an earlier oral treatment of less severe sepsis with a later treatment of more severe sepsis with parenteral antibiotic, which may not be directly comparable. However, for simplicity of messages the difference in administration of antibiotics was not taken into account.

Experts noticed that there would be neonates who would seek care, but wouldn't actually be able to access the level of care that they wanted to seek. Some of them are treated in the PATHS tool as those who did not get care. The experts questioned whether they should have been termed as “obtaining / not obtaining care” rather than “seeking / not seeking”? Given that this difference doesn’t mean anything to their outcome, we kept the terminology as “not seeking” for clarity, although the point is valid. Similarly, we termed some parents who sought care at the PHC level and were referred to SHC (but could not comply, although they probably wanted to) as “did not comply”, although the more correct term would be “could not comply”.

Experts commented that care seeking is likely to be greater in cases that are apparently more severe and asked whether this is somehow factored in the model. However, this notion would then need to be attributed to parents’ own diagnostic algorithms, which is captured in sensitivity and specificity of diagnosis at the home care level. The same is true at the CHW and PHC level.

Finally, experts suggested that there may be some hidden correlations or interactions between the parameters used in the model and wondered if eg, a 50% change in care seeking would need to be corrected for actual access, etc.? However, since all parameters in the tool are related to specific branches of the decision tree and each one can be modified, the users can account for these possible correlations wherever they use the tool and think that there is a reasonable case to do this.

### Agreed values for model parameters based on evidence and expert opinion

In this part of the paper, we will explain how we decided on the most likely value of the key model parameters, using both evidence and expert opinion, and being constrained by the global estimate of 907 000 neonatal deaths in 2008.

**Figure 2:**
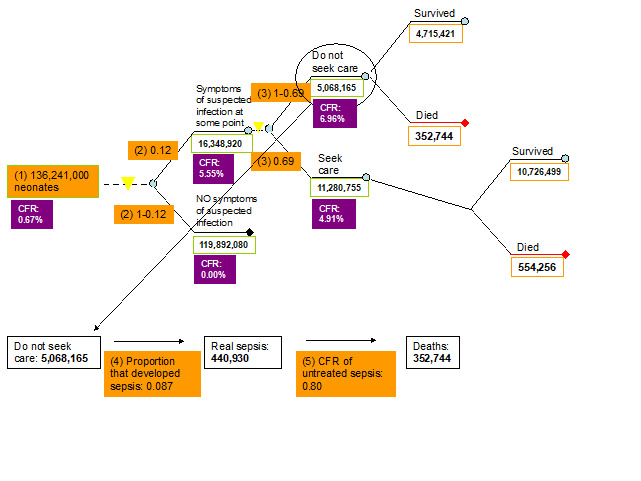
Parameters of the model relevant to the number of neonates each year globally, proportion of those who develop symptoms of suspected infection, care-seeking behaviour and case-fatality rates (CFRs) in different pathways. (CFR for “neonates” represents the absolute risk of dying during the first month of life; all other CFRs are case-fatality rates among neonates with suspected infections or real sepsis).

1. *Number of neonates.* Data for this model parameter were obtained from the World Health Organization’s (WHO) website [15]. The annual total number of neonates was estimated to be 136 241 000 and this estimate was based on the 2008 WHO data (**Figure 2**, **Table 1**).

2. *Proportion of neonates with symptoms of suspected infection.* The only estimate of larger geographic relevance that we could find was based on a review by Thaver and Zaidi published in 2009 [16]. Their paper reviewed studies published in the early 2000s and this parameter was estimated to equal about 0.17 [16]. However, most technical experts thought that this estimate would be too high globally for the year 2008. Therefore, we performed Delphi exercise and took the median of what the total of 12 experts reported (see their list in acknowledgement). The final value in our model was set to 0.12 (**Figure 2**, **Table 1**).

3a. *Proportion of neonates with symptoms of suspected infection that sought care.* Data for this indicator were reviewed and summarised by Chandran et al. [11] and Herbert et al. [12]. In total, 22 studies (18 from South-East Asia and 4 from Africa) were identified that reported care seeking behaviour for newborn infections. Care seeking outside of the home ranged from a lower bound of 0.10, an upper bound of 1 and a median of 0.69 (**Figure 2**, **Table 1**).

3b. *Proportion of neonates with symptoms of suspected infection that didn't seek care.* We reviewed the information for this indicator separately. However, the data assembled was of a much lesser quality compared to the data that were assembled for the indicator of proportion of neonates with symptoms of suspected infection that sought care. Therefore this indicator was set to be equal to 1 minus the proportion of neonates with symptoms of suspected infection that sought care (1 – 0.69 = 0.31) (**Figure 2**, **Table 1**).

**Figure 3:**
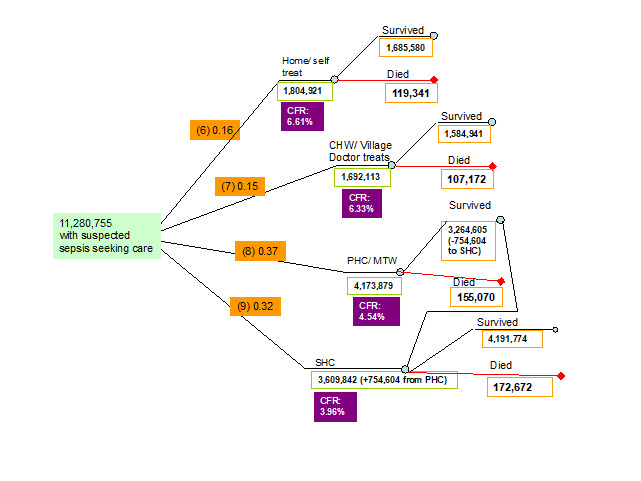
Parameters of the model relevant to the highest level of health system to which access has been realized for the neonates with suspected infection who sought care. CFR – case-fatality rate in each pathway.

**Figure 4:**
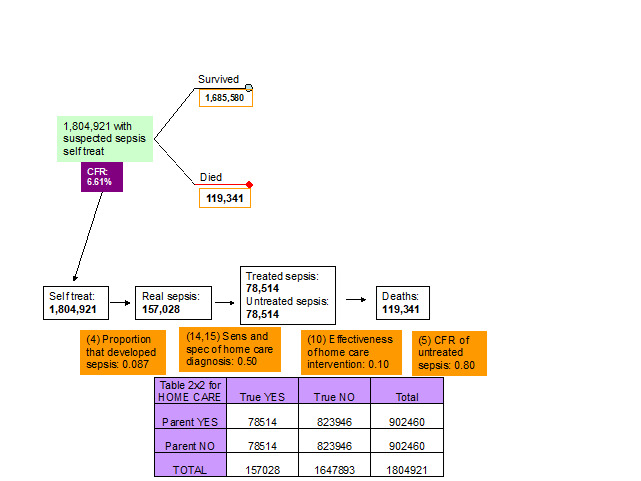
Parameters of the model relevant to the outcome of neonates with suspected infection whose parents applied self-treatment. CFR – case-fatality rate in each pathway.

**Figure 5:**
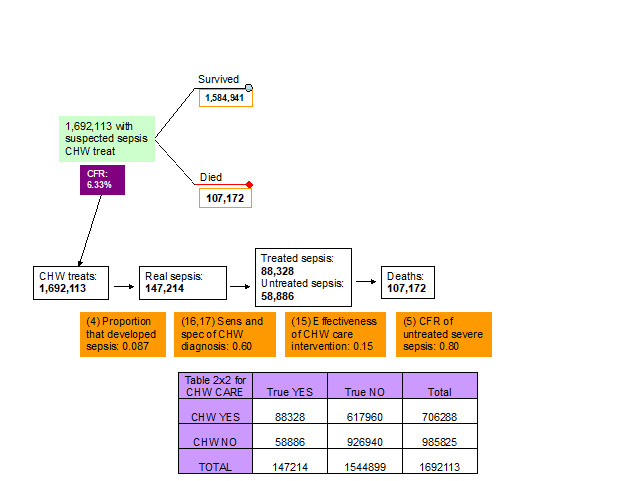
Parameters of the model relevant to the outcome of neonates with suspected infection who received some care from the local community health worker (CHW). CFR – case-fatality rate in each pathway.

4. *Proportion of neonates with symptoms of suspected infection that developed sepsis.* According to Thaver and Zaidi [16], 27.5% of neonates with suspected infection developed laboratory confirmed sepsis. However, the experts in the third TAG meeting thought that this estimate was too high and did not reflect the current situation. Based on the pneumonia model by Rudan et al. [13,14], 8.7% of pneumonia cases have severe disease and therefore we decided to use this estimate to reflect the amount of neonates with suspected infection that will develop sepsis (**Figure 2**, **Table 1**).

5. *Case fatality ratio of untreated sepsis.* There is no evidence of what the CFR of untreated neonatal sepsis is. Neonatal sepsis is a very severe disease and the experts in all three TAG meetings agreed that a CFR of 80% will reflect the current situation in low income settings (**Figure 2**, **Table 1**).

6. *Proportion of neonates with symptoms of suspected infection that sought care received treatment from their carers at home (self treatment).* Data for this indicator were reviewed and summarised by Chandran et al. [11] and Herbert et al. [12]. In total, 3 studies (2 studies from SE. Asia, 1 study from Africa) were identified that reported the proportion of neonates of those that sought care that received home treatment for newborn infections with a lower bound of 0.06, an upper bound of 0.37 and a median of 0.16 (**Figure 3**, **Table 1**).

7. *Proportion of neonates with symptoms of suspected infection that sought care and received treatment from community health workers (CHW treatment).* In total 4 studies, all from SE. Asia identified reported the proportion of caregivers who accepted care from CHWs for their ill neonates with a lower bound of 0.01 and an upper bound of 0.72. However, since these studies addressed care acceptance and not care seeking, this indicator was set to be equal to 1 minus the proportion of neonates that sought care at home, at a primary health care (PHC) facility and at a secondary health care (SHC) facility (1-0.16-0.37-0.32 = 0.15) (**Figure 3**, **Table 1**).

8. *Proportion of neonates with symptoms of suspected infection that sought care and received treatment in a primary health care facility (PHC treatment) or from a medically trained provider.* Data for this indicator were reviewed and summarised by Chandran et al. [11] and Herbert et al. [12]. In total 11 studies (9 studies from SE. Asia, 2 studies from Africa) were identified that reported the proportion of neonates of those that sought care that received treatment for newborn infections in a PHC facility with a lower bound of 0.04 and an upper bound of 0.83 and a median of 0.37 (**Figure 3**, **Table 1**).

9. *Proportion of neonates with symptoms of suspected infection that got treatment in a SHC treatment.* Data for this indicator were reviewed and summarised by Chandran et al. [11] and Herbert et al. [12]. In total 6 studies (5 studies from SE. Asia, 1 study from Africa) were identified that reported the proportion of neonates of those that sought care that received treatment for newborn infections in a SHC facility with a lower bound of 0.04 and an upper bound of 0.66 and a median of 0.32 (**Figure 3**, **Table 1**).

10. *Effectiveness of home care interventions.* Although it is unlikely that the home treatment interventions will have a major effect on neonatal sepsis (especially if they are based on traditional methods), the experts thought that the effectiveness of home treatment should be set equal to 0.10 as they may use antibiotics prescribed to their children previously that are still effective (**Figure 4**, **Table 1**).

11. *Effectiveness of CHW interventions.* The experts thought that the effectiveness of CHW treatment interventions will depend mainly on whether CHWs have access to antibiotics or not. However, they thought that access to antibiotics of CHWs is limited in the developing countries and therefore it is more likely that they will use traditional based methods. The consensus was that the effectiveness of the CHW interventions should be set to about 0.15 (**Figure 5**, **Table 1**).

12. *Effectiveness of PHC interventions.* In a review by Theodoratou et al. [17] we found that the effectiveness of antibiotics on childhood pneumonia in a community setting was 35% and after accounting for compliance this estimate was scaled up to 70%. We assumed, however, that the effectiveness of antibiotics for neonatal sepsis will be lower since: (i) neonatal sepsis is a more severe disease and (ii) the administration of antibiotics to neonates is more challenging, which could compromise their effectiveness. There are also issues related to access to correct antibiotics and antibiotic formulations and their availability in low-resource settings, that we reviewed separately in the paper by Lee et al. [18]. Therefore, after discussion with the experts at the third TAG meeting we set the effectiveness of PHC interventions to 0.35 (**Table 1**).

13. *Effectiveness of SHC interventions.* The experts thought that the effectiveness of the interventions used in a SHC facility will be a mixture of first- and second-line antibiotics depending on the setting and country. However, they thought that the effectiveness of the interventions in SHC facilities should be closer to the effectiveness of community administered antibiotics for pneumonia after accounting for compliance and this parameter was set equal to 0.60. Then, it was further revised to 0.58 in order to “fit” the total number of deaths in this model to the “envelope” of 907 000 defined by Black et al. [1]. This was the only parameter that was slightly altered in order to achieve the perfect fit of the number of deaths to the envelope (**Table 1**).

14-15. *Sensitivity and specificity of clinical algorithm of carer to detect sepsis at home care treatment.* The experts at the third TAG meeting decided that there was no evidence to suggest that the ability of the parents to recognise the symptoms of true sepsis was better than a random guess and therefore the sensitivity and specificity of the clinical algorithm that the parents used to decide whether to self treat the newborn or not were set equal to 0.50 and 0.50 respectively (**Figure 4**, **Table 1**).

16-17. *Sensitivity and specificity of clinical algorithm of CHW to detect sepsis.* The experts at the third TAG meeting decided that the ability of a CHW to diagnose sepsis was quite low, but still higher than the ability of the parents. Therefore, sensitivity and specificity of the clinical algorithm that the CHWs used to decide whether to treat the newborn or not were set equal to 0.60 and 0.60, respectively (**Figure 5**, **Table 1**).

**Figures 6:**
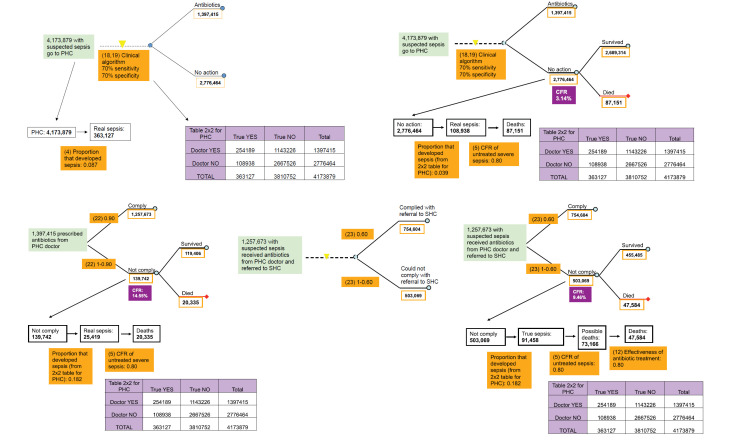
Parameters of the model relevant to the outcome of neonates with suspected infection who received care from a primary health care physician (PHC). CFR – case-fatality rate in each pathway; SHC – secondary health care.

18-19. *Sensitivity and specificity of clinical algorithm of medical doctor at PHC facility to detect sepsis.* The experts at the third TAG meeting agreed that the ability of a medical doctor to diagnose sepsis at PHC facility is better than the ability of CHW or parents and they thought that a sensitivity and specificity of 0.70 and 0.70 reflects the situation in 2010 (**Figure 6**, **Table 1**).

20-21. *Sensitivity and specificity of clinical algorithm at SHC to detect sepsis.* The experts at the third TAG meeting decided that the ability of a medical doctor to diagnose sepsis at a SHC facility is better than the ability of a medical doctor in a PHC (based on experience and number of severe cases that are reviewed in SHC facilities compared to PHC facilities) and they thought that a sensitivity and specificity of 0.90 and 0.70 reflects the situation in 2010 (**Figure 6**, **Table 1**).

22. *Compliance of parents with antibiotics prescription from PHC doctor.* The experts at the third TAG meeting suggested that the compliance of the parents with antibiotic prescription at a PHC facility will be high and the consensus was that this would be 0.90 (**Figure 6**, **Table 1**).

23. *Proportion of parents that comply with referral to SHC from PHC doctor.* The proportion of parents that will comply with a referral to SHC facility by a PHC doctor will depend on several factors including: (i) proximity or even existence of a SHC facility and (ii) ability (mainly financial) and willingness (determined mainly by social factors) of the parents to travel the distance between the PHC and SHC facilities. There was no hard evidence for this indicator and our estimate of 0.60 was based on the experts opinions mainly based on their experiences in the field (**Figure 6**, **Table 1**).

## RESULTS

**Figure 7:**
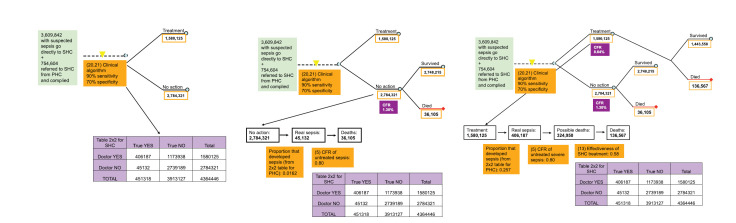
Parameters of the model relevant to the outcome of neonates with suspected infection who received care at secondary health care level (SHC). CFR – case-fatality rate in each pathway; SHC – secondary health care.

**Figure 8:**
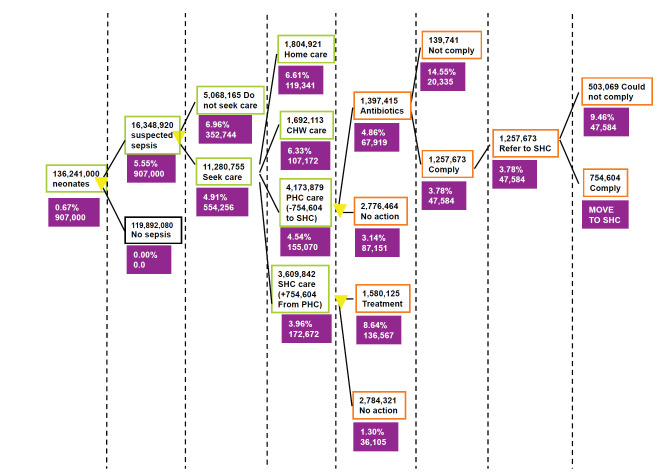
Pathways to deaths from neonatal infections globally: the basic model with the estimates of the number of neonates passing through each pathway (colour-bordered rectangles), and case fatality rate and the number of deaths in each pathway (violet rectangles). CFR – case-fatality rate; PHC – primary health care; SHC – secondary health care. *The denominator that was used for the CFR calculation did not include 1 597 335 neonates that after PHC treatment were referred and went to a SHC facility).

Based on the assumptions and values of the indicators we distributed the neonatal sepsis deaths to the branches of the decision tree presented in **Figure 7**. The number of deaths and CFRs for each step are presented in the decision tree of **Figure 8** and described in the section below. Based on our model the total number of neonatal sepsis deaths was estimated to be 907 000, to match the situation in 2008 as estimated by Black et al. [1]). **Figure 8** shows how case-fatality rates differ between different branches of the decision tree and along the different paths. Having information on the true status of a neonate at the various points of a decision tree, as indicated by yellow triangles, would allow carers to take the child along a different path that is associated with a lower case-fatality rate.

**Table 2** shows pathways to mortality from newborn sepsis based on the situation in 2008. A total of 352 744 newborns died because did not seek care and did not receive any treatment. Further 119 341 died although they sought care, but they were limited to self-treatment and did not respond well. Moreover, 107 172 died although they sought care, but CHW treated them and they did not respond. Further 87 151 died although they sought care at PHC level, but PHC doctor missed diagnosis and didn't treat. Based on the assumptions of the PATHS model, 20 335 died although they sought care and PHC doctor intended to treat them, but parents didn’t comply. Then, 47 584 died although they sought care and PHC doctor treated them, but parents couldn't go to seek care at the SHC level. It is also estimated that 36 105 died although sought care at SHC, but SHC doctor missed diagnosis and didn't treat. Finally, as many as 136 567 died although they sought care at SHC and SHC doctor treated them, but they did not respond well to treatment. All of these deaths add to 907 000, which was an estimate for the year 2008 provided by Black et al. [1].

**Table 2:** Pathways to mortality based on the situation in 2008

Pathways to mortality without any interventions (situation in 2008)	Deaths
Died because did not seek care and did not receive any treatment	352 744
Died although sought care, but self-treated and did not respond	119 341
Died although sought care, but CHW-treated and did not respond	107 172
Died although sought care at PHC, but PHC doctor missed diagnosis and didn't treat	87 151
Died although sought care and PHC doctor intended to treat but parents did not comply	20 335
Died although sought care and PHC doctor treated but parents couldn't go to SHC	47 584
Died although sought care at SHC, but SHC doctor missed diagnosis and did not treat	36 105
Died although sought care at SHC and SHC doctor treated, but did not respond	136 567
**Total deaths from newborn sepsis (2008)**	**907** **,000**

CHW – community health worker; PHC – primary health care physician; SHC – secondary health care

**Figure 8** summarises how these deaths are a consequence of varying case-fatality rates in different branches of the decision tree. From the health systems perspective, there are at least 8 possible points of intervention that are described in more detail in **Boxes 1** to **8**. Having a true information on the status of each newborn at the 8 apparent points of possible intervention, which are market with yellow triangles, would allow carers, CHWs and doctors to take the child along the path which is associated with a smaller CFR. This would change the fate for some of them and reduce the overall number of deaths, but it would also require a “near-perfect” diagnostic test at each level. It should also be realised that any estimate of “effectiveness” (or potential impact fraction) for the options 1-3 will be relevant to all 907 000 deaths in the world, but any estimate of “effectiveness” for the option 4-6 will only be relevant to a smaller number, which result from the branches following the possible point of intervention, while any estimate of “effectiveness” for the option 7 and 8 will be relevant only to those deaths that occur in SHC, reducing the potential impact fraction at the global level even further.

Box 1 **Active screening for bacterial infection by community health workers (CHWs) through population-based programmes**

TYPE OF TEST: predictive

WHAT DOES IT TEST FOR: bacterial infection in all newborns

PURPOSE OF INTRODUCING THE TEST: indication for early antibiotic treatment; prevention of progress to severe sepsis

SENSITIVITY REQUIREMENT: needs to be extremely sensitive to save lives by finding additional true cases, but also to prevent false negatives, who would normally seek care, from NOT seeking care because of the test result (“primum nil nocere”)

SPECIFICITY REQUIREMENT: nearly 100% (otherwise massive congestion of PHC and unnecessary antibiotic treatment)

LABORATORY REQUIREMENTS: none; must be handheld

COST: cost of the test itself must be extremely low, because hundreds of millions would be needed each year; cost of building the network of CHW would be extremely high

TRAINING NEEDS: cost of training in addition to clinical algorithm must be very low

FEASIBILITY OF SPECIMEN COLLECTION: good if saliva is target or test non-invasive worse if host response targeted; almost unfeasible if pathogen targeted; if test is conducted on a daily basis during a time window – risk of side-effects huge (eg, wound infections)

ACCEPTANCE OF FAMILIES TO TESTING: high if saliva; low if blood and multiple visits

COMPLIANCE OF IMPLEMENTOR WITH TEST RESULTS: What if no symptoms/need in clinical algorithm but, test positive? Specificity would need to be perfect (no false positives)

CHANGE IN CARE-SEEKING BEHAVIOUR: In what % of newborns who test positive, would parents change their decision and choose to seek / accept care? – this is where life-saving potential lies

HOW DOES IT SAVE LIVES: It would save lives mainly through change in care-seeking behaviour. It has a definite life-saving potential, but the costs and other test requirements do not make this option feasible anytime soon

Box 8 **Exact etiological diagnosis by a doctor at the secondary health care (SHC) facility after care has been sought.**

TYPE OF TEST: diagnostic

WHAT DOES IT TEST FOR: aetiology of infection and antibiotic resistance

PURPOSE OF INTRODUCING THE TEST: refining the hospital-based treatment (and surveillance for resistance?)

SENSITIVITY REQUIREMENT: needs to be extremely high because of cost implications

SPECIFICITY REQUIREMENT: needs to be extremely high because of cost implications (near 100%)

LABORATORY REQUIREMENTS: we may assume capacity

COST: test needs to be cheap; but if 2^nd^ line drug treatments are introduced, the costs will soar;

TRAINING NEEDS: larger than for all other tests

FEASIBILITY OF SPECIMEN COLLECTION: pathogens are targeted; therefore, uncontaminated sampling is critical – training needed

ACCEPTANCE OF FAMILIES TO TESTING: needs evaluation; it will be much better than for most other options

COMPLIANCE OF IMPLEMENTOR WITH TEST RESULTS:

- What if the test shows the need to change the standard treatment? Could they comply? Would they comply?

- Test can improve treatment effectiveness at the SHC level

CHANGE IN CARE-SEEKING BEHAVIOUR:

Not applicable at this level

HOW DOES IT SAVE LIVES: Primarily by changing the effectiveness of antibiotic treatment among cases of sepsis who reached SHC and received antibiotic treatment based on IMCI; test would need to be highly sensitive and specific, and it would have an appreciable life saving potential

Finally, to facilitate the implementation of the PATHS tool, we developed an addition to this article: an Excel file (Appendix S1 in the **Online Supplementary Document**) which contains all the information and parameters from this article. The users should be able to easily modify all the numbers and “tailor” the tool to their own context. We also filmed the video with a short demonstration of how this Excel sheet can be used and the parameters changed. The link to the video on YouTube is: https://www.youtube.com/watch?v=oK6z5nj3xqw&list=PLwE5KAphA-_EdRYyVaxVMHOzDEdf1sGLb&index=9 (it can also be found by searching for the Lecture 9 within the YouTube channel called “Global Health Economics and Priority Setting”).

## DISCUSSION

The PATHS tool is very useful in helping policy-makers to understand why, how, and where within the health system do the deaths occur and what can be done about this. The maximum potential of an intervention to remove burden will clearly be realised when the intervention can be applied at the community level. Other levels of intervention application could potentially have more impact if care-seeking behaviour could be improved and if access to antibiotics and health care could be improved. The situation at the time, in 2008, suggested that the more realistic the context for intervention implementation, the less opportunity for potential large impact of that intervention.

Even if, eg, absolutely perfect diagnostic tests were available (meaning that they are characterised by 100% sensitivity and specificity, extremely low cost, no laboratory requirements, easy to use), they would still have very limited effectiveness because of two other factors: (i) at the community level, the prognosis for those correctly diagnosed is still rather poor; the opportunities for saving lives are missed because they may not choose to comply (a need for education of parents), the community health workers are not allowed to treat (need for legislation change), the effectiveness of treatment by community health workers may not be high (need for training of CHWs), and the referral to PHC and SHC may be largely failing due to poverty and poor access (need for conditional cash transfers and improved access); and (ii) at the facility level, there are simply much fewer deaths to be averted, and they cluster more severe cases which still have high mortality regardless of the test, while the validity of clinical algorithm can’t be improved as much using the test.

In **Box 9**, we presented some illustrative examples of what can be achieved in terms of mortality reduction through application of the PATHS model. The tool predicts that an increase in care seeking from 69% to 100% would result in 103 731 lives saved, with a global potential impact fraction (PIF) of only 11.4%. This example shows how even universal health coverage does not necessarily lead to massive gains in child mortality reduction unless many other factors are also addressed. Another example shows that increasing specificity of all diagnostic tests to 100% would still not result in any lives saved, as specificity is not really important in the community setting. Improvements in sensitivity of diagnostics at various points, though, would lead to lives saved. An increase of sensitivity of diagnosis at home from 50% to 100% would save 6281 lives, which is a global PIF of 0.7%. At the CHW level, an increase in sensitivity from 60% to 100% would save 7066 lives, with global PIF of 0.8%. At the PHC, an increase in sensitivity from 70% to 100% could save 35 535 lives, with global PIF of 3.9%. Finally, at SHC level an increase in sensitivity from 90% to 100% would save 20 931 lives, with the global PIF of 2.3%. Then, if a compliance with a referral from PHC to SHC could increase from 60% to 100%, this would save 12 574 lives with a global PIF of 1.4%. If treatment effectiveness could be improved, then an increase from 35% to 100% at the PHC level would save 47 584 lives (global PIF of 5.3%), and at the SHC an increase of treatment effectiveness from 58% to 100% would save 136 567 lives (global PIF of 15.1%).

Box 9 **A summary on maximum potential impact at the global level of different improvements: impact on 907 000 deaths.**

Care seeking increases from 69% to 100%: 103 731 (potential impact fraction (PIF) = 11.4%)

Sensitivity 100% at home: 6281 lives saved (PIF = 0.7%)

Sensitivity 100% for CHWs: 7066 (PIF = 0.8%)

Sensitivity 100% at PHC: 35 535 (PIF = 3.9%)

Sensitivity 100% at SHC: 20 931 (PIF = 2.3%)

Referral from PHC to SHC from 60% to 100%: 12 574 (PIF = 1.4%)

Effectiveness of PHC treatment from 35% to 100%: 47 584 (PIF = 5.3%)

Effectiveness of SHC treatment from 58% to 100%: 136 567 (PIF = 15.1%)

(PIF – potential impact fraction; CHW – community health worker; PHC – primary health care physician; SHC – secondary health care; CFR – case-fatality rate)

Clearly, a combination of several improvements would have synergistic effects and lead to appreciable reduction in child mortality. There is no doubt that diagnostic tests could save many lives, and that they should continue to receive investments to improve their validity, deliverability and affordability. However, those investments should not be occurring in isolation from parallel investments in improving the 8 contexts described in **Boxes 1** to **8**, so that the tests could realize lot more of their potential. Some things that would help towards this end would include evaluating programs for education of parents to improve their compliance, promoting legislation change to empower CHWs to become an active part in this agenda, evaluating programs for training of CHWs to use tests and administer treatments correctly and improving care-seeking behaviour through programs of conditional cash transfers. Perhaps most importantly, improving access to PHC and SHC for everyone would be much desired, so that the majority of suspected infections are seen in the contexts where the difference between health systems’ intervention and non-intervention, and the resulting change in CFR, are the largest, as summarised in **Figure 8**.

There have been numerous attempts by international organizations and academia-based researchers to develop models that could use evidence to explain the “architecture” of the burden of disease and death, along with the effects of the key risk factors and the implemented interventions. Some of the most prominent ones were reviewed [19] and they include Lives Saved Tool [20], EQUIST Tool [21-23], One Health Tool [24], HipTool [25] or HealthCatalyst tool [26]. Still, none of them, in their current form, manage to visualise how the contexts of the local and national health systems determine the outcomes in a systematic way. The advantage of the proposed PATHS tool is that it integrates both the “horizontal” and the “vertical” component that need to be considered in understanding the causes of unwanted health outcomes. This allows policymakers to properly understand where the potential for saving lives truly lies. Given how many parameters need to be taken into account, this will rarely be intuitive and it does require a model like PATHS for more clarity.

To demonstrate the translational potential of this tool, we provided an Excel sheet which allows running the PATHS model (**Online Supplementary Document**). We also provided a video with a tutorial on how to use the PATHS tool. Although the illustrative example pertains to historic deaths from newborn sepsis in 2010, which were a really useful example to demonstrate the utility of the PATHS tool, we would like to stress that there is a large translational potential of this same decision tree model to other infectious diseases, populations and settings. Those new models would need to be appropriately adapted based on the disease of interest, population and setting and engage experts in respective fields to define the most plausible values for the parameters. However, this should not be a difficult process and the PATHS model should find its application in visualising and explaining the “architecture” of the morbidity and/or the mortality of any human disease in any population, from neonatal sepsis to chronic non-communicable diseases of late onset.

Box 2 **Diagnosis of bacterial infection by parents or community health workers (CHW) based on symptoms of possible infection**

TYPE OF TEST: diagnostic

WHAT DOES IT TEST FOR: bacterial infection in presence of symptoms of infection

PURPOSE OF INTRODUCING THE TEST: improvement of sensitivity and specificity of clinical algorithm; better indication for antibiotic treatment & reduction of unnecessary treatment

SENSITIVITY REQUIREMENT: must improve clinical algorithm + thermometer and be very sensitive – otherwise, low life saving potential and concern over growing false negatives who would normally seek care

SPECIFICITY REQUIREMENT: must improve clinical algorithm + thermometer and be very specific – otherwise, congestion of primary health care (PHC) and secondary health care (SHC)

LABORATORY REQUIREMENTS: none; must be handheld

COST: as low as possible

TRAINING NEEDS: minimal in addition to clinical algorithm for CHW; a campaign needed for parents; note low skill of implementors

FEASIBILITY OF SPECIMEN COLLECTION: not a problem if host response targeted; major problem if pathogens targeted

ACCEPTANCE OF FAMILIES TO TESTING: needs evaluation

COMPLIANCE OF IMPLEMENTOR WITH TEST RESULTS:

- What if symptoms present but test negative? How will that change their action?

- What if clinical algorithm shows no indication for treatment, but test positive? (needs evaluation)

CHANGE IN CARE-SEEKING BEHAVIOUR:

- How would confirmation of CHW’s suggestion for treatment by positive test / referral (or parents’ diagnosis of test positive) change their care-seeking behaviour? (needs evaluation)

- Danger: too high specificity will delay care-seeking for other problems that cause similar symptoms, eg, heart problem!

HOW DOES IT SAVE LIVES: It would save lives in several ways: (i) change in care-seeking behaviour; (ii) enrichment of true positive cases of sepsis among those who seek care; (iii) it would give powers to CHWs to treat neonatal infections; this test would have life-saving potential – requires attention

Box 3 **Diagnosis of severe infection by parents or community health workers (CHWs) based on symptoms of possible infection.**

TYPE OF TEST: diagnostic

WHAT DOES IT TEST FOR: whether there are signs of severe infection that requires referral to secondary health care (SHC) facility

PURPOSE OF INTRODUCING THE TEST: quick referral to SHC of newborns at high risk of death

SENSITIVITY REQUIREMENT: must improve clinical algorithm + thermometer and be very sensitive – otherwise, low life saving potential and concern over growing false negatives who would normally seek care

SPECIFICITY REQUIREMENT: must be close to 100% - otherwise massive congestion in SHC and risk of nosocomial infections

LABORATORY REQUIREMENTS: none; must be handheld

COST: as low as possible

TRAINING NEEDS: minimal in addition to clinical algorithm for CHW; a campaign needed for parents; note low skill of implementors

FEASIBILITY OF SPECIMEN COLLECTION: not a problem if host response targeted; major problem if pathogens targeted

ACCEPTANCE OF FAMILIES TO TESTING: needs evaluation

COMPLIANCE OF IMPLEMENTOR WITH TEST RESULTS:

- What if some symptoms of severe infection present but test negative? How will that change their action?

- What if clinical algorithm shows no indication for SHC referral, but test positive? (needs evaluation)

CHANGE IN CARE-SEEKING BEHAVIOUR:

- How would confirmation of CHW’s suggestion for referral to SCH by positive test / referral (or parents’ diagnosis of test positive) change their care-seeking behaviour? (needs evaluation)

HOW DOES IT SAVE LIVES: It would save lives in several ways: (i) change in care-seeking behaviour; (ii) enrichment of true positive cases of severe sepsis among those who seek care; this test would have life-saving potential – but requires access to SHC

Box 4 **Diagnosis of bact. Infection by a trained CHW/ doctor at the primary health care (PHC) facility after care has been sought.**

TYPE OF TEST: diagnostic

WHAT DOES IT TEST FOR: bacterial infection in presence of symptoms of infection

PURPOSE OF INTRODUCING THE TEST: improvement of sensitivity and specificity of clinical algorithm; better indication for antibiotic treatment and reduction of unnecessary treatment

SENSITIVITY REQUIREMENT: must improve clinical algorithm + thermometer by a lot to really make a difference

SPECIFICITY REQUIREMENT: doesn’t matter that much, unless we want to reduce the unnecessary antibiotic treatment

LABORATORY REQUIREMENTS: preferably none; we may assume some, but minimal capacity

COST: as low as possible

TRAINING NEEDS: minimal

FEASIBILITY OF SPECIMEN COLLECTION: not a problem if host response targeted; in theory, less of a problem than with CHW/parents if pathogens targeted

ACCEPTANCE OF FAMILIES TO TESTING: needs evaluation; it will be better than at options shown in **Boxes 1** to **3**

COMPLIANCE OF IMPLEMENTOR WITH TEST RESULTS:

- What if symptoms present (IMCI) but test negative? How will that change their action?

- What if clinical algorithm shows no indication for treatment, but test positive? (needs evaluation)

CHANGE IN CARE-SEEKING BEHAVIOUR:

- How would confirmation of PHC HW’s suggestion for treatment by positive test / referral change their care-seeking behaviour – eg, visit to secondary health care (SHC) facility? (needs evaluation)

- Danger: too high specificity will delay care-seeking for other problems that cause similar symptoms, eg, heart problem!

HOW DOES IT SAVE LIVES: It would save lives primarily by reducing the number of cases of true sepsis who reach PHC but never get antibiotic treatment because the doctor / trained HW misses the correct diagnosis due to imperfect IMCI algorithm; the test would need to be extremely sensitive to have potential to save many lives

Box 5 **Diagnosis of severe infection by a trained community health worker (CHW)/ doctor at the primary health care (PHC) facility after care has been sought.**

TYPE OF TEST: diagnostic

WHAT DOES IT TEST FOR: whether there are signs of severe infection that requires referral to secondary health care (SHC) facility

PURPOSE OF INTRODUCING THE TEST: quick referral to SHC of newborns at high risk of death

SENSITIVITY REQUIREMENT: must improve clinical algorithm + thermometer by a LOT to have effect – close to 100%

SPECIFICITY REQUIREMENT: must be close to 100% - otherwise massive congestion in SHC and risk of nosocomial infections

LABORATORY REQUIREMENTS: preferably none; we may assume some, but minimal capacity

COST: as low as possible

TRAINING NEEDS: minimal

FEASIBILITY OF SPECIMEN COLLECTION: not a problem if host response targeted; in theory, less of a problem than with CHW/parents if pathogens targeted

ACCEPTANCE OF FAMILIES TO TESTING: needs evaluation; it will be better than at options shown in **Boxes 1** to **3**

COMPLIANCE OF IMPLEMENTOR WITH TEST RESULTS:

- What if some symptoms of severe infection present but test negative? How will that change their action?

- What if clinical algorithm shows no indication for SHC referral, but test positive? (needs evaluation)

CHANGE IN CARE-SEEKING BEHAVIOUR:

- How would confirmation of PHC HW’s suggestion for referral to SCH by positive test / referral (or parents’ diagnosis of test positive) change their care-seeking behaviour? (needs evaluation)

HOW DOES IT SAVE LIVES: Primarily by reducing the number of cases of severe sepsis who reach PHC but never get referred to hospital - either because the doctor misses the true severity or because parents do not comply. The test would need to be extremely sensitive and specific, but it would have a considerable life saving potential

Box 6 **Exact etiological diagnosis by a trained community health care (CHW)/doctor at the primary health care (PHC) facility after care has been sought.**

TYPE OF TEST: diagnostic

WHAT DOES IT TEST FOR: aetiology of infection and antibiotic resistance

PURPOSE OF INTRODUCING THE TEST: refining the treatment (and surveillance for resistance?)

SENSITIVITY REQUIREMENT: needs to be very high because of cost implications (near 100%)

SPECIFICITY REQUIREMENT: needs to be maximized because of cost implications (near 100%)

LABORATORY REQUIREMENTS: preferably none; we may assume some, but minimal capacity

COST: test needs to be cheap; but if 2^nd^ line drug treatments are introduced, the costs will soar;

TRAINING NEEDS: much larger than for all other tests (OPTIONS 1-5)

FEASIBILITY OF SPECIMEN COLLECTION: pathogens are targeted; therefore, uncontaminated sampling is critical – training needed

ACCEPTANCE OF FAMILIES TO TESTING: needs evaluation; it will be better than at options shown in **Boxes 1** to **3**

COMPLIANCE OF IMPLEMENTOR WITH TEST RESULTS:

- What if the test shows the need to change the standard treatment? Could they comply? Would they comply?

- Test can improve treatment, or also trigger referral to secondary health care (SHC) if antibiotic resistance or dangerous pathogen (needs evaluation)

CHANGE IN CARE-SEEKING BEHAVIOUR:

- How does this change? (needs evaluation)

HOW DOES IT SAVE LIVES: Primarily by changing the effectiveness of antibiotic treatment among cases of sepsis who reached PHC and received antibiotic treatment based on IMCI; test would need to be highly sensitive and specific, and it would have a considerable life-saving potential

Box 7 **Diagnosis of bacterial infection by a doctor at the secondary health care (SHC) facility after care has been sought.**

TYPE OF TEST: diagnostic

WHAT DOES IT TEST FOR: bacterial infection in presence of symptoms of infection

PURPOSE OF INTRODUCING THE TEST: improvement of sensitivity and specificity of clinical guidelines (pocketbook); better indication for antibiotic treatment & reduction of unnecessary treatment

SENSITIVITY REQUIREMENT: must improve clinical algorithm (pocketbook) + thermometer

SPECIFICITY REQUIREMENT: must improve clinical algorithm (pocketbook) + thermometer

LABORATORY REQUIREMENTS: moderate (to good) capacity

COST: as low as possible

TRAINING NEEDS: considerable (clinicians and laboratory staff)

FEASIBILITY OF SPECIMEN COLLECTION: not a problem if host response targeted; less of a problem than OPTIONS 1-6 if pathogens targeted

ACCEPTANCE OF FAMILIES TO TESTING: needs evaluation; it will be very high

COMPLIANCE OF IMPLEMENTOR WITH TEST RESULTS:

- What if symptoms present (IMCI) but test negative? How will that change their action?

- What if clinical algorithm shows no indication for treatment, but test positive? (needs evaluation)

CHANGE IN CARE-SEEKING BEHAVIOUR:

Not relevant for this option

HOW DOES IT SAVE LIVES: It would save lives primarily by reducing the number of cases of true sepsis who reach SHC but never get antibiotic treatment because the doctor misses the correct diagnosis due to imperfect clinical algorithm; the test would need to be extremely sensitive and specific to have potential to save lives

## Additional material

Online Supplementary Document

## References

[1] Black RE Cousens S Johnson HL Lawn JE Rudan I Bassani DG Global, regional, and national causes of child mortality in 2008: a systematic analysis. Lancet. 2010;375:1969-87. 10.1016/S0140-6736(10)60549-1 20466419

[2] Darmstadt GL Batra M Zaidi AKM Parenteral antibiotics for the treatment of serious neonatal bacterial infections in developing country settings. Pediatr Infect Dis J. 2009;28(1 Suppl):S37-42. 10.1097/INF.0b013e31819588c3 19106762

[3] Bhutta ZA Zaidi AKM Thaver D Humayun Q Johnson HL Ali S Darmstadt GL Management of newborn infections in primary care settings: a review of the evidence and implications for policy? Pediatr Infect Dis J. 2009;28(1 Suppl):S22-30. 10.1097/INF.0b013e31819588ac 19106759

[4] Darmstadt GL Saha SK Ahmed ASMNU Chowdhury MAKA Law PA Ahmed S Alam MA Effect of topical treatment with skin barrier-enhancing emollients on nosocomial infections in preterm infants in Bangladesh: a randomised controlled trial. Lancet. 2005;365:1039-45. 10.1016/S0140-6736(05)71140-5 15781099

[5] Lawn JE Cousens S Zupan J Lancet Neonatal Survival Steering Team 4 million neonatal deaths: when? Where? Why? Lancet. 2005;365:891-900. 10.1016/S0140-6736(05)71048-5 15752534

[6] Bahl R Martines J Ali N Bhan MK Carlo W Chan KY Darmstadt GL Research priorities to reduce global mortality from newborn infections by 2015. Pediatr Infect Dis J. 2009;28(1 Suppl):S43-8. 10.1097/INF.0b013e31819588d7 19106763

[7] Ng PC Diagnostic markers of infection in neonates. Arch Dis Child Fetal Neonatal Ed. 2004;89:F229-35. 10.1136/adc.2002.023838 15102726 PMC1721679

[8] Lim YW Reducing the global burden of acute lower respiratory infections in children: the contribution of new diagnostics. Nature. 2006;444(Suppl. 1):9-18. 10.1038/nature05442 17159890

[9] Baqui AH Arifeen SE Williams EK Ahmed S Mannan I Begum N Seraji HR Effectiveness of home-based management of newborn infections by community health workers in rural Bangladesh. Pediatr Infect Dis J. 2009;28:304-10. 10.1097/INF.0b013e31819069e8 19289979 PMC2929171

[10] Darmstadt GL Oral antibiotics in the management of serious neonatal bacterial infections in developing country communities. Pediatr Infect Dis J. 2009;28(1 Suppl):S31-6. 10.1097/INF.0b013e3181958794 19106761

[11] Chandran A Herbert HK Lee AC Rudan I Baqui AH Assessment of the proportion of neonates and children in low and middle income countries with access to a healthcare facility: A systematic review. BMC Res Notes. 2011;4:536. 10.1186/1756-0500-4-536 22166258 PMC3253732

[12] Herbert HK Lee AC Chandran A Rudan I Baqui AH Care seeking for neonatal illness in low- and middle-income countries: a systematic review. PLoS Med. 2012;9:e1001183. 10.1371/journal.pmed.1001183 22412355 PMC3295826

[13] Rudan I Tomaskovic L Boschi-Pinto C Campbell H Global estimate of the incidence of clinical pneumonia among children under five years of age. Bull World Health Organ. 2004;82:895-903. 15654403 PMC2623105

[14] Rudan I Boschi-Pinto C Biloglav Z Mulholland K Campbell H Epidemiology and etiology of childhood pneumonia. Bull World Health Organ. 2008;86:408-16. 10.2471/BLT.07.048769 18545744 PMC2647437

[15] www.who.int; accessed on September 20th, 2011.

[16] Thaver D Zaidi AK Burden of neonatal infections in developing countries: a review of evidence from community-based studies. Pediatr Infect Dis J. 2009;28:S3-9. 10.1097/INF.0b013e3181958755 19106760

[17] Theodoratou E Al Jilaihawi S Woodward F Ferguson J Jhass A Balliet M The effect of case management on childhood pneumonia mortality in developing countries. Int J Epidemiol. 2010;39 Suppl 1:i155-71. 10.1093/ije/dyq032 20348118 PMC2845871

[18] Lee AC Chandran A Herbert HK Kozuki N Markell P Shah R Treatment of infections in young infants in low- and middle-income countries: a systematic review and meta-analysis of frontline health worker diagnosis and antibiotic access. PLoS Med. 2014;11:e1001741. 10.1371/journal.pmed.1001741 25314011 PMC4196753

[19] Rudan I Kapiriri L Tomlinson M Balliet M Cohen B Chopra M Evidence-based priority setting for health care and research: tools to support policy in maternal, neonatal, and child health in Africa. PLoS Med. 2010;7:e1000308. 10.1371/journal.pmed.1000308 20644640 PMC2903581

[20] The Lives Saved ToolAvailable: https://www.livessavedtool.org/. Accessed: 15 January 2020.

[21] Chopra M Campbell H Rudan I Understanding the determinants of the complex interplay between cost-effectiveness and equitable impact in maternal and child mortality reduction. J Glob Health. 2012;2:010406. 10.7189/jogh.01.010406 23198135 PMC3484756

[22] Waters D Theodoratou E Campbell H Rudan I Chopra M Optimizing community case management strategies to achieve equitable reduction of childhood pneumonia mortality: An application of Equitable Impact Sensitive Tool (EQUIST) in five low- and middle-income countries. J Glob Health. 2012;2:020402. 10.7189/jogh.02.020402 23289077 PMC3529311

[23] EQUISTAvailable: https://www.equist.info/. Accessed: 15 January 2020.

[24] OneHealth ToolAvailable: https://www.who.int/choice/onehealthtool/en/. Accessed: 15 January 2020.

[25] Health Interventions Prioritization ToolAvailable: http://hiptool.org/. Accessed: 15 January 2020.

[26] Health CatalystAvailable: https://www.healthcatalyst.com/. Accessed: 15 January 2020.

